# Could Eosinophilia predict clinical severity in nasal polyps?

**DOI:** 10.1186/s40248-017-0102-7

**Published:** 2017-08-21

**Authors:** Figen Aslan, Eren Altun, Serpil Paksoy, Gulay Turan

**Affiliations:** Balıkesir Universty School of medicine, Pathology Department, Balıkesir, Turkey

**Keywords:** Nasal polyps, Chronic rhinosinusitis, Eosinophils, Eosinophilia

## Abstract

**Background:**

Although nasal polyps are one of the most frequent diseases, their etiopathogenesis remains unclear.Since eosinophils are the main inflammatory cells in the substantial proportion of nasal polyp tissues, they are considered potentially responsible for the etiopathogenesis and prognosis of the disease. Aim of this study was to investigate the relation between mucosal and peripheral eosinophilia and their relation with disease severity in nasal polyps.

**Methods:**

The study included 53 patients with nasal polyps who underwent endoscopic sinus surgery. Preoperative Lund-MacKay computed tomography (CT) scores and the Lund-Kennedy endoscopic scores of the patients were recorded. Nasal polyp tissues were stained with hematoxylin and eosin, eosinophil counts were performed using high-power field (HPF, 400×) under the light microscope, and the patients were grouped as those with high mucosal eosinophil count and those with low mucosal eosinophil count.

**Results:**

The mean Lund-MacKay CT score and the mean Lund-Kennedy endoscopic score were higher in the patients with high mucosal eosinophil count than in those with low mucosal eosinophil count. Likewise, the mean Lund-MacKay CT score and the mean Lund-Kennedy endoscopic scores were significantly higher in the patients with high peripheral eosinophil count than in those with low peripheral eosinophil count (*p* < 0.05 for both). Moreover, the mean peripheral eosinophil count was significantly higher in the patients with high mucosal eosinophil count than in those with low mucosal eosinophil count (*p* < 0.05).

**Conclusion:**

Mucosal and peripheral eosinophilia can be used as a marker to predict disease severity in nasal polyps.

## Background

Nasal polyps are epithelial and stromal non-neoplastic proliferations of the nasal cavity and paranasal sinuses [1]. Despite many theories propounded regarding the etiopathogenesis of nasal polyps, it has not been clarified yet. Histologically, nasal polyps have myxoid and edematous stroma covered by respiratory epithelium exhibiting hyperplasia or squamous metaplasia and infiltrated predominantly by eosinophils [2, 3].

Eosinophils contain leukotrienes, eosinophilic cationic protein, major basic protein, platelet-activating factor, eosinophilic peroxidases, and other vasoactive substances that cause mucosal damage. These may play a critical role in the development of nasal polyps [4]. In addition to the studies confirming the relation between mucosal eosinophilia and disease severity, there are also studies defending just the opposite [4–6]. In addition, there is controversy regarding whether mucosal eosinophilia needs to be examined in nasal polyps and whether tissue eosinophil count needs to be reported in pathology reports or whether this is a waste of time and labor.

The present retrospective study aimed to investigate the relation between mucosal and peripheral eosinophilia and their relation with disease severity in nasal polyps.

## Methods

The present retrospective study included 53 patients who underwent endoscopic sinus surgery for nasal polyps. The patients did not receive topical or systemic corticosteroid or antibiotic therapy for at least 4 weeks prior to the surgery. Patients younger than 18 years of age were not enrolled in the study to exclude allergic fungal sinusitis, tumors, and cystic fibrosis.

Preoperative Lund-MacKay computed tomography (CT) scores and Lund-Kennedy endoscopic scores, which indicate disease severity, were obtained from the medical files of the patients. In the Lund-Mackay CT scoring system, total score ranges between 0 and 24 [7, 8]. Polyps were scored according to the Lund-Kennedy endoscopic scoring system [9] between 0 and 2 points, where a score of 0 indicates absence of polyps, a score of 1 indicates polyps in the middle meatus only, and a score of 2 indicates polyps beyond the middle meatus. Total score is between 0 and 4 for both sides.

The sections obtained from the paraffin blocks of surgical materials, which were fixed with formaldehyde, were stained using hematoxylin and eosin. In these sections, mucosal eosinophil count was performed using high-power field (HPF, 400×) in the area with the highest eosinophil density by three different pathologists. The arithmetic mean of the counts measured by the three pathologists was calculated. The patients were then grouped according to the mucosal eosinophil count as follows: patients with high mucosal eosinophil count (a count of >10 eosinophils/HPF) and patients with low mucosal eosinophil count (a count of ≤10 eosinophils/HPF) [7, 8]. Data regarding peripheral eosinophils were obtained from the patients’ medical files.

The relationship of Lund-MacKay CT score and the Lund-Kennedy endoscopic score with high and low mucosal eosinophilia and with high and low peripheral eosinophilia was investigated. In addition, whether there was a relationship between mucosal eosinophilia and peripheral eosinophilia was statistically analyzed.

### Statistical analysis

Data analyses were performed using the IBM SPSS Statistics for Windows (version 22.0; IBM Corp., Armonk, NY, USA). Descriptive statistics were expressed as number, percentage, and mean ± standard deviation, where appropriate. Two groups comparisons were performed using the Mann-Whitney U test for non-normally distributed continuous variables. Chi-square test was used for the comparisons of categorical variables. The relation between continuous variables was evaluated using Pearson’s correlation analysis. A *p* <0.05 was considered statistically significant.

## Results

The mean age of 53 patients with nasal polyps, out of whom 36 (67.9%) were male and 17 (32.1%) were female, was 47.2 ± 15.2 years. Out of the patients, 33 (62.3%) had a mucosal eosinophil count of >10/HPF (high mucosal eosinophil count), whereas 20 (37.7%) had a mucosal eosinophil count of ≤10/HPF (low mucosal eosinophil count). The mean preoperative Lund–Mackay CT score was 10.7 ± 1.1 and the mean Lund–Kennedy endoscopic score was 2.4 ± 1.16. The mean peripheral eosinophil count in the whole study group was 0.4 ± 0.3 × 10^3^/μL. The demographic characteristics, disease severity, and eosinophil values of the patients are shown in Table 1.

**Table 1:** Characteristics of the study population

Characteristics	Patients (*n* = 53)
Gender	
Females	17 (32.1)
Males	36 (67.9)
Age, year	47.2 ± 15.2
Disease severity	
Lund-MacKay CT score	10.7 ± 1.1
Lund-Kennedy endoscopic score	2.4 ± 1.1
Eosinophil values	
High mucosal eosinophil count (>10 eosinophils/HPF)	33 (62.3)
Low mucosal eosinophil count (≤10 eosinophils/HPF)	20 (37.7)
Peripheral eosinophil count (×10^3^/μL)	0.4 ± 0.3

Data are presented as mean ± standard deviation of number (%), where appropriate

*CT* computed tomography, *HPF* high-power field (400×)

A comparison of patients having a high mucosal eosinophil count (>10 eosinophil/HPF) and those having a low mucosal eosinophil count (≤10 eosinophil/HPF) revealed that the Lund–MacKay CT score, Lund–Kennedy endoscopic score, and peripheral eosinophil count (×10^3^/μL) were significantly higher in patients with a high mucosal eosinophil count (*p* = 0.001, for each; Table 2).

**Table 2:** Comparison of the patients with high and low mucosal eosinophil counts in terms of Lund-MacKay computed tomography score, Lund-Kennedy endoscopic score and peripheral eosinophil count

	Patients with high mucosal eosinophil count	Patients with low mucosal eosinophil count	
	(>10 eosinophil/HPF) Mean ± SD	(≤10 eosinophil/HPF) Mean ± SD	*p*
Lund-MacKay CT score	13.5 ± 4.9	6.2 ± 3.5	0.001
Lund-Kennedy endoscopic score	2.8 ± 1.1	1.7 ± 0.9	0.001
Peripheral eosinophil count (×10^3^/μL)	0.5 ± 0.3	0.1 ± 0.1	0.001

*HPF* high power field, *SD* standard deviation, *CT* computed tomography

The mean Lund–MacKay CT score and the mean Lund–Kennedy endoscopic score were significantly higher in patients with a high peripheral eosinophil count than in patients with a low peripheral eosinophil count (*p* = 0.001 and *p* = 0.002, respectively; Table 3).

**Table 3:** The Lund-MacKay computed tomography score and the Lund-Kennedy endoscopic score in patients with high and low peripheral eosinophil counts

	Lund-MacKay CT score Mean ± SD	Lund-Kennedy endoscopic score Mean ± SD
Patients with		
High peripheral eosinophil count	12.4 ± 5.5	2.8 ± 1.0
Low peripheral eosinophil count	7.2 ± 4.2	1.7 ± 0.9
*p*	**0.001**	**0.002**

*SD* standard deviation, *CT* computed tomography

In the correlation analyses, the peripheral eosinophil counts were found to be significantly correlated with the Lund–Mackay CT and Lund–Kennedy endoscopic scores in patients with nasal polyps (*r* = 0.353, *p* = 0.010 and *r* = 0.444, *p* = 0.001, respectively). The findings obtained from the correlation analyses are presented in Table 4.

**Table 4:** Findings obtained from the correlation analyses

		Lund-MacKay CT score	Lund-Kennedy endoscopic score
Peripheral eosinophil counts	*r*	0.353	0.444
	*p*	0.010	0.01

## Discussion

The pathophysiology of chronic rhinosinusitis (CRS) and nasal polyps remains unclear; however, eosinophilic inflammation has been reported to play a critical role. Histological studies have demonstrated high levels of eosinophils in nasal polyp tissues [8, 10–13]. In the present study, 62.3% of our patients with nasal polyps had high mucosal eosinophilia (mucosal eosinophilic inflammation defined as an eosinophil count of >10/HPF). Likewise, previous studies have demonstrated that sinonasal tissue in CRS with nasal polyps (CRSwNP) frequently exhibits tissue eosinophilia [5, 7, 8].

In general, CRS is classified according to the presence of accompanying nasal polyps. The recurrence rate in CRSwNP is higher despite successful surgical intervention and it requires a longer period of medical treatment. There are studies confirming the relation of mucosal eosinophilia with postoperative recurrence and disease severity in nasal polyps [4, 6, 12, 14]. This raises the question of whether the presence of nasal polyps or eosinophilia is more important in the classification of CRS.

Although the etiology of nasal polyposis is unknown in the majority of patients, etiological cause has been identified in very few patients. Polyps are encountered in many patients with aspirin sensitivity or allergic fungal sinusitis. Nasal polyps also occur frequently in patients with Kartagener’s syndrome [15].

Recently, outcomes suggesting a relation between bronchial asthma and chronic sinusitis have been reported. Both these conditions have similar pathogenesis indicating mucosal sensitivity against chronic stimulus. In addition, eosinophilic inflammation plays an important role in the pathogenesis of both diseases as the main source of chronic stimulus [4]. Although allergy used to be considered the etiology of nasal polyps, some opposite hypotheses have been propounded in studies conducted afterwards. It has been observed that patients with nasal polyps are non-atopic rather than atopic, and multiple positive skin test results are less common in nasal polyp patients than in the general population [15, 16]. In the present study, patients with allergic fungal sinusitis, those with cystic fibrosis, those with aspirin sensitivity, and those with asthma were not enrolled.

Steroid therapy and endoscopic sinus surgery are the most common therapeutic methods used for the treatment of nasal polyps. Steroids have been reported to be the most effective method among medical therapies [3, 4, 17]. Steroids relieve symptoms likely by downregulating the production and expression of cytokines, such as interleukin-5, that reduces eosinophil count [15]. Patients who were treated with antimicrobial agents or steroids within 4 weeks prior to endoscopic sinus surgery were not included in the present study.

In their study, Nakayama et al. [6] divided patients into four groups as follows: those having eosinophilic CRS (ECRS) with nasal polyps (ECRSwNP), those having ECRS without nasal polyps (ECRS without NP), those having non-ECRS with nasal polyps (NECRSwNP), and those having non-ECRS without nasal polyps (NECRS without NP). They determined a significantly higher recurrence rate in patients with mucosal eosinophilia, regardless of the presence or absence of nasal polyps. The prognosis was better in the NECRSwNP and NECRS without NP groups than in the ECRSwNP group. Accordingly, the authors concluded that mucosal eosinophilia is a more critical factor than nasal polyps in the classification of CRS.

Although the diagnosis of CRS is established according to the onset and duration of symptoms, many clinicians use CT scanning to verify the diagnosis, to assess disease severity, and to decide on the treatment method. Some systems are appropriate for the staging of disease severity. The Lund–Mackay staging system was developed as a simple assessment system to enable the choice of treatment [18–20]. Similar to the findings of previous studies, the present study determined that the mean Lund–Mackay CT and mean Lund–Kennedy endoscopic scores were significantly higher in patients with a high mucosal eosinophil count (13.5 ± 4.9 and 2.8 ± 1.1, respectively) than in those with a low mucosal eosinophil count (6.2 ± 3.5 and 1.7 ± 0.9, respectively) (*p* = 0.001 for both).

Similar to the results of the present study, in their study conducted on patients with CRS, Soler et al. [21] demonstrated the relation of mucosal eosinophilia with CT, endoscopy, and Smell Identification Test scores, which indicate disease severity. Moreover, they failed to determine a similar relationship with any of the other histological markers of inflammation investigated in their study. In a subsequent study, Soler et al. [7] measured the preoperative and postoperative quality of life (QOL) in patients with CRS using the Chronic Sinusitis Survey, the Rhinosinusitis Disability Index, and the Short Form-36 General Health Survey. They compared the improvements in QOL between patients with and without mucosal eosinophilia. As a result, they determined that patients without mucosal eosinophilia or polyps had the greatest improvement in QOL, whereas the patients with mucosal eosinophilia without polyps had the lowest improvement in QOL. Consequently, that particular study supported the need for CRS to be classified according to the presence or absence of mucosal eosinophilia.

In a study in which a tissue eosinophil count of >10/HPF was histopathologically defined as ECRS, Snidvang et al. [8] compared non-ECRS patients with ECRS patients and demonstrated that endoscopic and CT scores were more severe in the ECRS patients. Based on the study outcomes, they concluded that tissue eosinophilia could be a good marker of CRS, regardless of its subtypes.

Biopsy reports after endoscopic sinus surgery are usually limited to a general pathological diagnosis indicating chronic inflammation and excluding malignancy. In fact, our opinion is that a detailed and standardized definition of inflammation, particularly in terms of the presence or absence of eosinophilia, would provide a prognostic marker differentiating ECRS from non-ECRS. However, how tissue eosinophilia would be calculated and which value would be accepted as the upper limit might be another matter of debate. There are many studies wherein tissue eosinophilia calculation methods and limit values of tissue eosinophilia differ [mucosal eosinophilia ≥70 eosinophils/HPF [6], >5eosinophils/1HPF [21, 22], >10 eosinophils/HPF [7, 8].

In general, the blood eosinophil level is considered as a marker of eosinophilic tissue inflammation. Accordingly, studies have been conducted concerning the peripheral blood eosinophil count in nasal polyps and CRS [5, 8, 10, 22–25]. In the present study, the correlation analyses revealed that the peripheral eosinophil count was significantly and positively correlated with the Lund–Mackay CT and Lund–Kennedy endoscopy scores (*r* = 0.353, *p* = 0.01 and *r* = 0.444, *p* = 0.01, respectively).

Similar to the findings of the present study, Bryson et al. [10] demonstrated that the tissue eosinophil count tended to increase with an increasing disease severity in CRS patients with or without nasal polyps and they suggested a relation between blood eosinophil count and disease severity.

In the study by Ten Brinke et al. [24], asthmatic patients with extensive sinus disease were determined to have higher median serum eosinophil levels than those with limited sinus disease. Moreover, they found that eosinophils in peripheral blood were significantly and positively correlated with CT scores (*r* = 0.46, *p* < 0.001).

Kountakis et al. [22] found a correlation between peripheral eosinophil count and preoperative CT scores (*r* = 0.78, *p* < 0.05), as well as between peripheral eosinophilia and endoscopic scores (*r* = 0.40, *p* < 0.05). Moreover, they reported a correlation between mucosal eosinophil count and peripheral eosinophil count (*r* = 0.50, *p* < 0.05).

Snidvongs et al. [8] conducted a study of 51 patients with CRS and determined a correlation between tissue and serum eosinophilia (*r* = 0.33, *p* = 0.03). In the present study, the mean peripheral eosinophil count was significantly higher in patients with a high mucosal eosinophil count than in those with a low mucosal eosinophil count (*p* = 0.001).

Eosinophilic CRS has been accepted as a subgroup of CRSwNP since 2001 in Japan [23]. Peripheral eosinophilia is the characteristic blood sign of ECRS, and it is significantly associated with peripheral eosinophil count. For this reason, peripheral eosinophilia has a diagnostic importance for ECRS [23].

In a recent study conducted on severe CRS patients who had high CT scores, multiple prior surgeries, severe asthma, and extensive medication usage [5], it was reported that disease severity was not correlated with eosinophilia (>10 eosinophils/HPF) and there was no significant correlation between the absolute tissue eosinophil count and the Lund–Mackay CT score. Moreover, although they reported a significant difference among the asthmatic patients grouped according to their disease severity in terms of tissue eosinophil level, the level of mucosal eosinophilia did not significantly differ between patients with and without asthma. In addition, contrary to what expected, the absolute tissue eosinophil count was found not correlated with blood eosinophil count in that particular study, the results of which were opposite of both the present study and earlier studies, which might be the result of corticosteroid usage due to severe CRS in the majority of patients. In addition, the migration and activation of eosinophils may result in a significant tissue effect, even at low blood levels [5].

## Conclusion

Although the results of many studies are contrary to those herein, the findings of the present study revealed the positive correlation of the clinical severity of nasal polyps with peripheral and mucosal eosinophilia. In addition, before sinus surgery, peripheral eosinophilia may indicate a more extensive form of mucosal disease and can be used as a marker of mucosal eosinophilia. However, there is controversy regarding whether the tissue eosinophil count needs to be reported in pathology reports or whether this is a waste of time and labor. We are of the opinion that eosinophilia criteria must be identified and standardized before mucosal and peripheral eosinophilia can be used as prognostic information.

## References

[1] Mills SE. Nose, paranasal sinuses, and nasopharynx. In: Mills SE, Greenson JK, Hornick JL, Longacre TA, Reuter VE, editors. Stenberg’s diagnostic surgical pathology, vol. 952: Wolters Kluwer Health; 2015.

[2] Couto LGF, Fernades AM, Brandão DF, Santi Neto D, Valera FCP, Anselmo-Lima WT (2008). Histological aspects of rhinosinusal polyps. Braz J Otorhinolaryngol.

[3] Jankowski R, Bouchoua F, Coffinet L, Vignaud JM (2002). Clinical factors influencing the eosinophil infiltration of nasal polyps. Rhinology.

[4] Tosun F, Arslan HH, Karslioglu Y, Deveci MS, Durmaz A (2010). Relationship between postoperative recurrence rate and eosinophil density of nasal polyps. Ann Otol Rhinol Laryngol.

[5] Gitomer SA, Fountain CR, Kingdom TT, Getz AE, Sillau SH, Katial RK, et al. Clinical examination of tissue eosinophilia in patients with chronic rhinosinusitis and nasal polyposis. Otolaryngol Head Neck Surg. 2016;155:173–8. 10.1177/0194599816637856 26980909

[6] Nakayama T, Yoshikawa M, Asaka D, Okushi T, Matsuwaki Y, Otori N, et al. Mucosal eosinophilia and recurrence of nasal polyps-new classification of chronic rhinosinusitis. Rhinology. 2011;49:392–6. 10.4193/Rhino10.261 21991563

[7] Soler ZM, Sauer D, Mace J, Smith TL (2010). Impact of mucosal eosinophilia and nasal polyposis on quality-of life outcomes after sinus surgery. Otolaryngol Head Neck Surg.

[8] Snidvongs K, Lam M, Sacks R, Earls P, Kalish L, Philips S (2012). Structured histopathology profiling of chronic rhinosinusitis in routine practice. Int Forum Allergy Rhinol.

[9] Lund V, Kennedy D (1997). Staging for rhinosinusitis. Otolaryngol Head Neck Surg.

[10] Bryson JM, Tasca RA, Rowe-Jones JM (2003). Local and systemic eosinophilia in patients undergoing endoscopic sinus surgery for chronic rhinosinusitis with and without polyposis. Clin Otolaryngol.

[11] Morinaka S, Nakamura H (2000). Inflammatory cells in nasal mucosa and nasal polyps. Auris Nasus Larynx.

[12] Lackner A, Raggam RB, Stammberger H, Beham A, Braun H, Kleinhappl B (2007). The role of interleukin-16 in eosinophilic chronic rhinosinusitis. Eur Arch Otorhinolaryngol.

[13] Ishitoya J, Sakuma Y, Tsukuda M (2010). Eosinophilic chronic rhinosinusitis in Japan. Allergol Int.

[14] Gun T, Demir M, Gur OE, Ozel D, Dogru H, Boztepe OF (2016). A novel predictive marker for the recurrence of nasal polyposis following endoscopic sinus surgery. Eur Arch Otorhinolaryngol.

[15] Mygind N, Dahl R, Bachert C (2000). Nasal polyposis, eosinophil dominated inflammation, and allergy. Thorax.

[16] Bachert C, Gevaert P, Holtappels G, Johansson SGO, Cauwenberge P (2001). Total and specific IgE in nasal polyps is related to local eosinophilic inflammation. J Allergy Clin Immunol.

[17] Burgel PR, Cardell LO, Ueki IF, Nadel JA. Intranasal steroids decrease eosinophils but not mucous expression in nasal polyps. Eur Respir J. 2004;24:594–600. 10.1183/09031936.04.00014404 15459138

[18] Lund VJ, Mackay IS (1993). Staging in rhinosinusitis. Rhinology.

[19] Polzehl D, Moeller P, Riechelmann H, Perner S (2006). Distinct features of chronic rhinosinusitis with and without nasal polyps. Allergy.

[20] Hopkins C, Browne JP, Slack R, Lund V, Brown P (2007). The Lund-Mackay staging system for chronic rhinosinusitis: how is it used and what does it predict?. Otolaryngol Head Neck Surg.

[21] Soler ZM, Sauer DA, Mace J, Smith TL (2009). Relationship between clinical measures and histopathologic findings in chronic rhinosinusitis. Otolaryngol Head Neck Surg.

[22] Kountakis SE, Arango P, Bradley D, Wade ZK, Borish L (2004). Molecular and cellular staging for the severity of chronic rhinosinusitis. Laryngoscope.

[23] Sakuma Y, Ishitoya J, Komatsu M, Shiono O, Hirama M, Yamashita Y (2011). New clinical diagnostic criteria for eosinophilic chronic rhinosinusitis. Auris Nasus Larynx.

[24] Ten Brinke A, Grootendorst DC, Schmidt JT, De Bruine FT, van Buchem MA, Sterk PJ (2002). Chronic sinusitis in severe asthma is related to sputum eosinophilia. J Allergy Clin Immunol.

[25] Wei JL, Kita H, Sherris DA, Kern EB, Weaver A, Ponikau JU (2003). The chemotactic behavior of eosinophils in patients with chronic rhinosinusitis. Laryngoscope.

